# Randomness as a behavioral marker of attentional control during the attention training technique and its neural correlates

**DOI:** 10.3389/fpsyt.2026.1765858

**Published:** 2026-04-24

**Authors:** Kristina Schwarz, Julia Lützkendorf, Franziska Giller, Tanja Endrass

**Affiliations:** Department of Clinical Psychology and Psychotherapy, Technische Universität Dresden, Dresden, Germany

**Keywords:** Attention Training Technique (ATT), attentional control, dual-task, fMRI, random number generation

## Abstract

**Background:**

The Attention Training Technique (ATT) is a core intervention of metacognitive therapy (MCT) effective in reducing repetitive negative thinking by engaging the lateral prefrontal cortex (PFC). The current study tests whether a parallel random number generation (RNG) task can serve as a behavioral marker of attentional engagement.

**Methods:**

We conducted two studies using the fMRI-adapted ATT paradigm combined with a parallel RNG task. In the behavioral study (N = 36), we compared two task versions with short (21 presses) versus long (45 presses) trials. In the fMRI study (N = 42), the ATT paradigm was performed with either parallel RNG or a motor control task. Markers of randomness (i.e., Redundancy, Repetition Gap, and Coupon scores) were compared between ATT conditions. Trait AC was measured using the Attentional Control Scale (ACS).

**Results:**

Randomness was reduced during ATT compared to control trials in both studies (*p* <.021). Additional analyses showed that 1) across conditions, two of the RNG scores correlated with indices from a standard RNG task (*p* <.029); 2) ATT-control differences in RNG performance were detected in short and long trials of the ATT paradigm; and 3) ATT-control differences in Redundancy were associated with self-reported trait AC (*p* = .039). Further, RNG selectively activated regions within the dorsal attention network (*p_FWE_* <.05) and dual-task interference attenuated ATT-related task-network activation.

**Conclusions:**

These findings establish RNG as a feasible and sensitive behavioral marker of ATT engagement and offer the opportunity to link behavioral performance during ATT to individual differences in AC. However, dual-task demands limit the use of RNG as a behavioral marker, particularly in fMRI which calls for alternative, behavior-independent measures of attentional effort.

## 1 Introduction

Attentional control (AC) is a core cognitive capacity that allows individuals to flexibly select, shift, and broaden the focus of attention according to situational demands. Converging evidence suggests that reduced AC is a transdiagnostic vulnerability factor for emotional disorders, including depression and anxiety (1, 2). Such deficits are strongly associated with repetitive negative thinking styles, such as worry and rumination, which maintain and exacerbate psychopathology (3, 4). Within the self-regulatory executive function model (5), these maladaptive thinking styles are conceptualized as part of a Cognitive Attentional Syndrome (CAS). The CAS involves perseverative self-focused attention, threat monitoring, and unhelpful coping strategies. Crucially, the model posits that CAS activity is sustained by dysfunctional top-down executive control, reflecting both maladaptive beliefs about control strategies (e.g., that worry is uncontrollable, but useful in preventing negative outcomes) and voluntary attentional processes (e.g., repeatedly directing attention toward perceived threats), which together maintain maladaptive processing patterns (6). To counter these maladaptations, metacognitive therapy (MCT) introduces interventions aimed at strengthening metacognitive regulation. A central method is the Attention Training Technique (ATT) (7), a structured auditory exercise designed to improve attentional flexibility and weaken self-focused processing. ATT consists of three subcomponents: selective attention to a single sound, rapid switching between sounds, and divided attention to multiple sounds simultaneously. It is assumed to operate through repeated practice, which trains flexible allocation of attention and rapid disengagement from self-focused processing, thereby weakening perseverative patterns central to the CAS and challenging metacognitive beliefs such as uncontrollability (e.g., by providing experiential evidence that attention can be flexibly regulated). Regular practice of ATT reduces rumination and worry and has demonstrated clinical efficacy both as part of MCT and as a stand-alone intervention (8, 9). Given its clinical impact and strong theoretical foundation, neuroscientific research has increasingly sought to investigate the neural mechanisms underlying ATT.

### 1.1 The neural correlates of ATT

Findings across modalities converge on the involvement of large-scale control networks, particularly the fronto-parietal control network (FPN). This network comprises lateral and dorsomedial prefrontal as well as parietal regions, which are densely interconnected and coordinate executive control across tasks and domains (10, 11). According to Cole and colleagues (12), FPN efficiency represents a transdiagnostic resilience factor, enabling individuals to flexibly recruit executive strategies and buffer against symptoms. Alterations in FPN functioning have been linked to impaired AC and psychopathology across disorders (13, 14).

Several studies suggest that ATT modulates neural processes within the FPN and related networks. For example, EEG and fNIRS studies report increased control network activity during ATT compared to passive listening (15, 16), consistent with heightened engagement of top-down AC processes during task execution. Further, fMRI work has shown altered connectivity patterns in depression (17, 18) and reduced FPN engagement during emotional distraction after ATT training (19). The latter potentially reflects reduced CAS-related processing of threat, leading to less interference from emotionally salient stimuli and, consequently, a lower demand for top-down control. However, the number of studies assessing the neural correlates during the execution of ATT is limited. Existing studies vary considerably in control conditions, ranging from simple rest periods (20) to passive listening (17), which limits comparability and interpretability. To overcome these challenges, we recently developed an fMRI-adapted ATT paradigm that directly contrasts ATT performance in two components of the ATT (i.e., focused attention and rapid switching) with perceptually matched passive listening conditions (21). This approach allowed us to isolate neural processes during ATT while controlling for basic auditory stimulation. Across two independent samples, the paradigm elicited robust activation of the FPN, particularly in the inferior and middle frontal cortex, supporting its suitability for investigating the neurocognitive mechanisms of ATT. However, a key limitation of this paradigm is the absence of objective behavioral performance markers that quantify the AC demands of the ATT task. In clinical practice, ATT progress is typically indexed by patients’ subjective self-focus ratings, which serve as a surface indicator of CAS activity (7). While we similarly collected trial-wise ratings of self-focus and effort which differentiate between ATT and control conditions, these measures are self-reported and potentially biased. Without an independent performance-based marker, the degree of actual engagement with ATT instructions remains difficult to quantify, which constrains both experimental control and the potential to assess training- and psychotherapy-induced alterations.

### 1.2 Random number generation and its neural correlates

A potential candidate for such a performance marker is to combine ATT with a second task, random number generation (RNG). RNG requires participants to produce sequences of digits that approximate random output while inhibiting habitual counting routines (22, 23). Successful performance requires continuous executive engagement, including the updating of previously used numbers, inhibition of habitual strategies, monitoring of responses, and flexible shifting between strategies (24, 25). RNG has thus been established as a sensitive probe of AC (26, 27) and has proven useful in detecting performance impairments in disorders associated with frontal dysfunction, such as Alzheimer’s disease, schizophrenia, or dementia (28–35).

Neuroimaging studies further support RNG as a valid marker of executive control. Across methodologies, RNG consistently recruits prefrontal and parietal regions, overlapping with the FPN. In particular, the lateral prefrontal cortex is reliably activated during RNG tasks using PET (36–38), fMRI (39), fNIRS (40, 41), and EEG (42, 43). Mechanistically, the dorsolateral prefrontal cortex (DLPFC) is thought to suppress stereotyped (i.e. non-random) responses (36), which collapses under increasing cognitive load, leading to reduced prefrontal activity alongside diminished randomness (37, 39). Causal manipulations using TMS over the DLPFC reduce randomness by impairing the inhibition of prepotent counting strategies (36, 38). Collectively, these findings indicate that RNG reliably recruits dorsolateral prefrontal brain regions critical for AC.

RNG is particularly well suited for dual-task paradigms in which two attention-demanding activities compete for shared resources. When executive control is limited, performance declines in both tasks (44). A selective reduction in randomness in the RNG under higher task demands, as compared to a low-demand control condition, provides a useful index of AC requirements of a specific task. Accordingly, RNG has frequently been combined with secondary tasks such as oddball detection, card sorting, visual tracking, or random movement generation (43–48). Such studies consistently demonstrate that RNG performance declines under increased executive load, tapping specifically into updating, inhibition, and set-shifting functions (47, 48). Interestingly, two studies used a parallel RNG task to examine the effects of rumination, both showing that randomness declined under induced rumination in patients with generalized anxiety disorder and in individuals with high trait anxiety (49, 50). This likely reflects that rumination consumes executive resources thereby reducing the ability to inhibit prepotent responses and generate random output. This underlines the sensitivity of RNG to subtle variations in executive demands, making it a promising behavioral marker of AC in the ATT paradigm.

### 1.3 Present study and hypotheses

We conducted two complementary studies – one behavioral and one during functional MRI – in which participants completed our recently developed ATT paradigm (21) with a parallel RNG task. We pursued the following aims. First, we tested the hypothesis that randomness would be lower during ATT trials compared to control trials. We examined this in the two independent samples to allow replication. Second, we explored 1) associations between RNG markers obtained from the dual-task ATT paradigm and from a standard RNG task, 2) differences between RNG markers in two versions of the dual-task ATT paradigm with varying trial lengths, and 3) associations between RNG markers and self-reported trait AC. Third, we investigated the neural correlates of the dual-task ATT paradigm, expecting that random vs. single button presses will engage DLPFC activation. Given that ATT engaged more ventral parts of the PFC (16, 21), we expect that the dual RNG task will not fully mask task-specific neural activation in the ATT paradigm. Further, we test whether previously detected brain-behavior links between ATT-related PFC activation and self-report AC (21) would diminish under dual-task conditions.

## 2 Materials and methods

All scripts and main data that support the findings of this study are available in the Open Science Framework (OSF) at https://osf.io/7q25a/overview.

### 2.1 Sample

In total, 86 healthy individuals participated in this study. These participants were recruited sequentially to take part in two separate studies. Six participants had to be excluded from analyses due to extreme RNG markers (i.e., values > 3 interquartile ranges from the upper or lower quartile; *n* = 5 in sample 1 and *n* = 1 in sample 2) and two due to excessive head motion (> 0.5 mm mean frame-wise displacement and/or in over 20% of all acquired frames (FD) (51) in the fMRI sample. This resulted in 36 participants in study one (behavioral) and 42 participants in study two (fMRI) – see **Table 1** for more information.

**Table 1:** Sample description.

	Sample 1 (*n* = 36) Behavioral		Sample 2 (*n* = 42) MRI	
Sex (m/f/d)	5/31/0		16/26/0	

	Mean	SD	Mean	SD
Age	23.40	5.00	27.40	7.70
CAS sum scores	56.04	15.91	54.78	15.02
ACS total	51.72	7.42	53.31	8.21

SD, standard deviations; ACS, Attentional Control Scale (52). CAS, Cognitive Attentional Syndrome Scale.

Participants were recruited via public advertisement. Inclusion was limited to individuals without current psychological disorders, as assessed by a structured telephone interview using the SCID-5 screening module. None of the participants reported current psychotherapeutic treatment or the use of psychoactive medication. Additional exclusion criteria were (1) prior experience with ATT training, (2) fMRI contraindications, (3) past or present neurological disorders, cardiovascular disease, or head trauma, and (4) hearing disabilities. All participants provided written informed consent, and the procedures were approved by the local Ethics Committee (SR-EK-151032023).

### 2.2 ATT paradigm and dual-task extensions

#### 2.2.1 Overall study description

We conducted two studies: one behavioral and one fMRI study. In Study 1 (behavioral), participants completed two runs of the dual-task ATT–RNG paradigm with different trial lengths (Run 1: 34 s; Run 2: 72 s) to examine whether RNG performance within the ATT paradigm depends on the number of generated responses.

**Figure 1:**
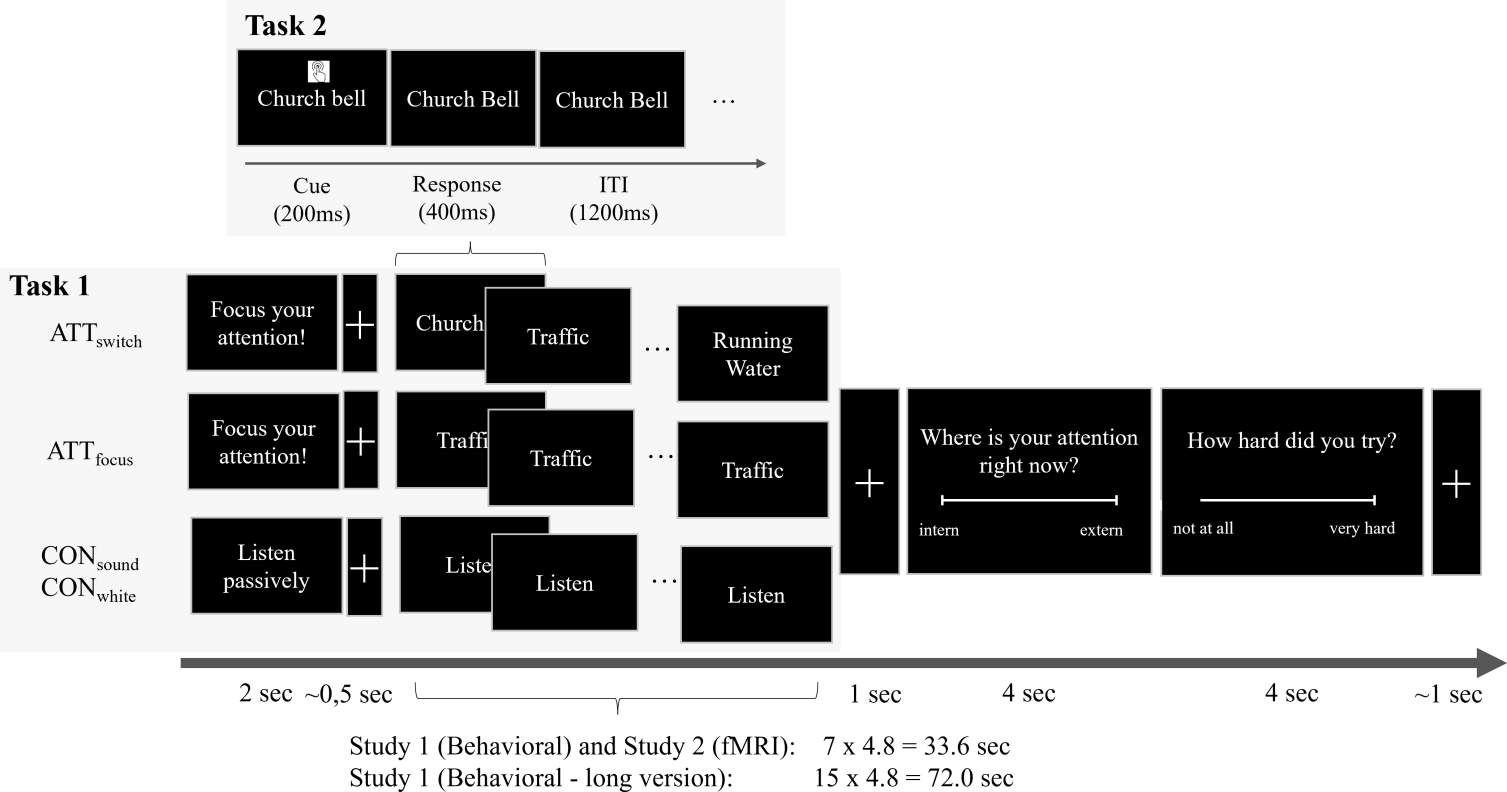
Task strucutre of the ATT paradigm [Task 1, adapted from Schwarz et al., (21)] with a parallel RNG task (Task 2). Task structure and timing. RNG, Random Number Generation; ATT, Attention Training Technique condition; CON, control condition; ATT_switch_, switch the attentional focus rapidly between sounds; ATT_focus_, focus attention to a specific sound for a longer period of time; CON_white_, Passively listen to white noise (low-level control); CON_sound_, Passively listen to alternative sounds .Touch icon: Touch icons created by https://www.flaticon.com/free-icons/touch.

In Study 2 (fMRI), participants completed the ATT paradigm under three conditions: (1) without a concurrent task [data reported in Sample 1 in Schwarz et al. (21)], (2) with a parallel RNG task, and (3) with a parallel simple key-press task (**Figure 1**, **Table 2**).

**Table 2:** Overview of the two complementary studies.

Study	RNG task	RNG: Key presses per trial	ATT: Conditions	Nr. of trials	Duration (min)
Study 1 (Behavioral)	RNG short	21	ATT: Switch, Focus CON: White, Sounds	4*8	24.3
	RNG long	45	ATT: Switch CON: White	2*8	21.3
Study 2 fMRI	ATT only	–	ATT: Switch, Focus CON: White, Sounds	4*4	12.4
	RNG short	21		4*4	12.4
	Simple key pressing	21		4*4	12.4

RNG, Random Number Generation task; Nr., Number; min, minutes; CON, control.

#### 2.2.2 ATT task

We adapted the ATT paradigm used previously (21) by adding a parallel dual RNG or simple key-press task (**Figure 1**). In the ATT paradigm, participants listened either to standardized ATT sounds (clock ticking, church bell, bird song, traffic), already used in prior ATT research (http://www.metakognitivetherapie.de/) (16, 21, 53), or to control sounds (**Figure 1**) presented via high-quality noise-cancellation headphones. The clinical version of ATT usually consists of a continuous audio sequence of roughly 12 minutes, comprising three phases: about 6 minutes of sustained selective attention to a single sound, 4 minutes of quickly shifting attention between different sounds, and 2 minutes of divided attention across multiple sounds at once. Following previous studies (16, 21) we modified this structure to suit neuroimaging requirements by implementing discrete, clearly separated blocks. Since the divided attention component represents the shortest segment of the training, and to keep the task manageable while ensuring a sufficient number of trials, this condition was excluded. The paradigm therefore concentrates on rapid switching (ATT_switch_) and selective focusing (ATT_focus_) which were compared to two passive listening control conditions (CON_sounds_ and CON_white_). Depending on the condition, participants were instructed to either focus on a single sound throughout the trial (ATT_focus_), switch between different sounds every 4 seconds (ATT_switch_), or simply listen in a relaxed manner to alternative sounds (CON_sounds_) or white noise (CON_white_). Each trial began with a 2-s centrally displayed instruction cue indicating the upcoming condition. Subsequently, the audio stream started and the presentation of a centrally displayed word indicated the current attentional focus (ATT conditions) or passive listening (control condition). In the ATT_switch_ condition, the instruction word changed every 4 s. Words and conditions were presented in a pseudorandomized order. After each trial, participants rated the current direction of their attention (internal vs. external) and the effort invested in the previous trial (low to high) using a five-point Likert scale (**Figure 1**). The exact number and duration of trials slightly differed between studies (see **Table 2** and **Figure 1** for details).

To ensure that the task instructions were fully understood and correctly implemented, participants first completed a brief practice session (n = 4 trials) prior to the main experiment. In addition, they were asked to verbally explain the task instructions to the experimenter to confirm comprehension.

#### 2.2.3 Dual RNG-task

Participants performed the RNG task continuously and concurrently throughout each ATT trial. During each trial (approximately 30–70 s, depending on the study), a visual cue appeared above the centrally displayed ATT instruction word at intervals of approximately 1.6 seconds, signaling participants to press a key randomly using any of their ten fingers (see Task 2 in **Figure 1**). Participants were instructed to respond quickly but without following any deliberate or systematic pattern. If they fell out of rhythm, they were asked to briefly pause and then continue. In Study 1 (behavioral), participants used a standard keyboard, whereas in Study 2 (fMRI), they held one MRI-compatible keyboard in each hand to enable the use of all ten fingers. To ensure task comprehension and familiarization, participants first completed a short practice RNG sequence, followed by one practice run of the ATT paradigm with the parallel RNG task (see Task 2 in **Figure 1**).

#### 2.2.4 Dual simple key-pressing task

In Study 2 (fMRI), participants additionally performed a third run in which a simple key press task was done in parallel to the ATT task. In this version, participants were asked to press a button with the left index finger in response to the displayed cue. The setup of the task was identical to the dual RNG task (see **Table 2**, **Figure 1**).

### 2.3 Calculation of RNG measures

Implementing RNG within a trial-based ATT paradigm poses challenges for quantifying randomness. Standard RNG protocols use single long sequences (i.e. > 100 responses), whereas dual-task versions, like ours, yield multiple shorter sequences that are averaged across conditions. This requires measures that remain sensitive with shorter response sets. Recent investigations (54) recommend three indices for such sequences—Redundancy, Repetition Gap, and Coupon score—which capture complementary aspects of randomness and allow for robust assessment even under these constrained conditions.

#### 2.3.1 Redundancy

Redundancy quantifies the uneven distribution of response alternatives, with higher values indicating greater repetition and thus lower randomness.


$$Redundancy=100*(1-\frac{log_{2}(n)-\frac{1}{n}(\sum a_{i}*log_{2}(a_{i}))}{log_{2}(a)})$$

Here, *n* denotes the sequence length, *a* is the total number of response alternatives, and *a_i_* is the number of occurrences of the *i*-th response alternative. Higher Redundancy indicate a concentration on specific response alternatives, reflecting lower randomness performance.

#### 2.3.2 Repetition Gap

Repetition Gap quantifies the average spacing between successive repetitions of the same response, with higher values indicating that repeated responses are more widely spaced and thus reflect higher randomness.


$$Repetition\;Gap=\frac{1}{N_{r}}\sum_{i=1}^{N_{r}}Gap_{i}$$

Here, Gap*_i_* ​ denotes the number of items between the *i*-th repetition of a given response, and N*_r_*​ is the total number of repeated responses in the sequence. Higher Repetition Gap values indicate that repeated responses are spaced further apart, reflecting higher randomness performance compared to lower values. The possible range of Repetition Gap depends on the sequence length and the distribution of repeated responses.

#### 2.3.3 Coupon score

Coupon Scores quantify how broadly participants sampled the response options within each trial, we computed the coupon score representing the average number of responses required until all options had occurred at least once. To account for minor differences in sequence length due to missing values, coupon length was divided by the total number of responses in the trial. In the classical approach, incomplete sequences (i.e. sequences where not all response options were used) are assigned a fixed value of *n + 1* (with *n* = total number of response options). Given our relatively short sequences (*n* ≈ 20 vs. *n* ≈ 100 in typical implementations), this method would produce a high rate of complete coupons and substantial ceiling effects. To avoid this problem, we implemented a gradual adjustment that scales with the degree of incompleteness, using


$$L_{adjusted}=L+\;\frac{nOptions-k}{nOptions}$$

where *L* is the observed coupon length and *k* the number of unique options observed. This approach distinguishes between strongly repetitive sequences (e.g., “11111111”) and nearly complete ones (e.g., “1234567888”), yielding a more sensitive and continuous measure of how comprehensively participants sampled the available response options. Overall, higher Coupon Scores indicate that participants repeated responses before all options occurred at least once, reflecting lower randomness performance.

### 2.4 Analyses of RNG differences across ATT conditions

RNG scores were calculated based on the sequences produced for each trial and averaged across trials within each condition. To test for differences between conditions, repeated-measures ANOVAs were conducted in SPSS (IBM, SPSS, version 29). In case of significant effects, *post-hoc* tests were used to further investigate differences between conditions. Analyses were performed separately in both samples to assess the replicability of the results. For study 1 (behavioral), we used the parameters of the short version (i.e. 21 button presses), to ensure comparability between the two samples and because the long version only included two of the four conditions (see **Table 2**). Significance was set to.05, using Bonferroni-Holm correction for the three RNG indices.

### 2.5 Analyses of influencing factors

#### 2.5.1 Association to state-of-the art RNG task performance

To adapt the RNG task as a parallel task to the ATT paradigm, several modifications to the original task were necessary (e.g., shorter sequences, averaging across multiple trials, etc.). To test whether aggregated RNG scores are associated with standard RNG performance (23), participants in a subsample (Study 1: *n* = 31; Study 2: *n* = 21), additionally performed a classical RNG task, where they generated a sequence of 100 numbers with the same pace as in the ATT paradigm (i.e. 1.6 seconds). We calculated the same RNG indices (i.e., Redundancy, Repetition Gap, and Coupon score) and correlated it with the average RNG scores calculated in the control conditions of the ATT paradigm using Pearson correlation.

#### 2.5.2 Effect of sequence length

We tested whether differences in randomness between ATT and control condition depend on the length of the trials by using repeated measures ANOVAs with trial length (short: 21 button presses vs. long: 45 button presses) and condition (ATT_switch_ vs. CON_white_) as within factors (see **Table 2**).

#### 2.5.3 Association with trait AC

We used the Attentional Control Scale (ACS) (52), a 20-items questionnaire to assess trait AC, which we previously linked to the neural correlates of ATT (21). In order to investigate whether trait AC was related to differences in RNG markers between ATT and control conditions, we used repeated measures ANCOVAs for each of the RNG markers and included trait AC as covariate of interest. To increase statistical power, we combined the samples of both studies for the analyses and included a covariate of no interest for study. Questionnaire data were not available for 2 subjects, leaving *n* = 77 datasets in these analyses.

### 2.6 fMRI parameters and analyses

Functional (TR: 869 ms, TE: 38 ms, flip angle 58°, FOV: 264 mm, and 60 axial slices, voxel size: 2.4 mm³) and structural (TR: 2, 000 ms, TE: 1.97 ms, flip angle 8°, FOV: 256 mm, 208 slice, voxel size: 1 mm³) images were obtained on a 3 Tesla Trio Siemens scanner. Standard preprocessing was performed in SPM12 (http://www.fil.ion.ucl.ac.uk/spm/), including slice-time correction, realignment to the mean image using field map correction, indirect normalization and spatial smoothing (8mm).

At the first-level, task regressors for the ATT conditions (ATT_switch_ and ATT_focus_) and control conditions (CON_sounds_, CON_white_) were entered separately for the two runs (RNG vs. single button press) into one general linear model (GLM) together with regressors for the instruction and rating periods and the six motion parameters as nuisance regressors. To investigate the functional activation during ATT compared to control conditions; and RNG compared to single button press, individual contrast estimates (1. ATT > CON and 2. RNG > single) were subjected to separate second-level one-sample t-tests. To investigate the interaction between condition and task run, an interaction contrast (single (ATT > CON) > RNG (CON > ATT) was defined and subjected to a second-level one-sample t-test. To probe brain-behavior association between ATT-related brain responses in the lateral PFC and trait AC, the sum score of the ACS was included as an additional regressor in second-level analyses.

Significance was assessed at the voxel-level and defined *a priori* as *p* <.05, family-wise error (FWE) corrected within the whole brain. In line with our previous study (21) we additionally used regions-of-interest analyses using a combined mask comprising the prefrontal parts of the right and left FPN as defined by Glasser et al. (52) (see **Supplementary Figure 1**).

## 3 Results

### 3.1 RNG Scores in ATT vs. control conditions

Descriptive results of the RNG markers are depicted in **Table 3** and correlational results in **Supplementary Table 1**.

**Table 3:** Descriptive results of randomness marker.

Randomness marker	Sample 1 (*n* = 36) behavioral		Sample 2 (*n* = 42) fMRI	
	Mean	SD	Mean	SD
Redundancy				
ATT	11.46	3.66	10.02	3.25
CON	9.75	4.02	8.55	3.32
Repetition Gap				
ATT	5.93	.71	6.28	.67
CON	6.36	.74	6.51	.66
Coupon Score				
ATT	1.13	.04	1.10	.04
CON	1.11	.05	1.08	.05

SD, standard deviation. Randomness marker calculated based on (26).

#### 3.1.1 Redundancy

**Figure 2:**
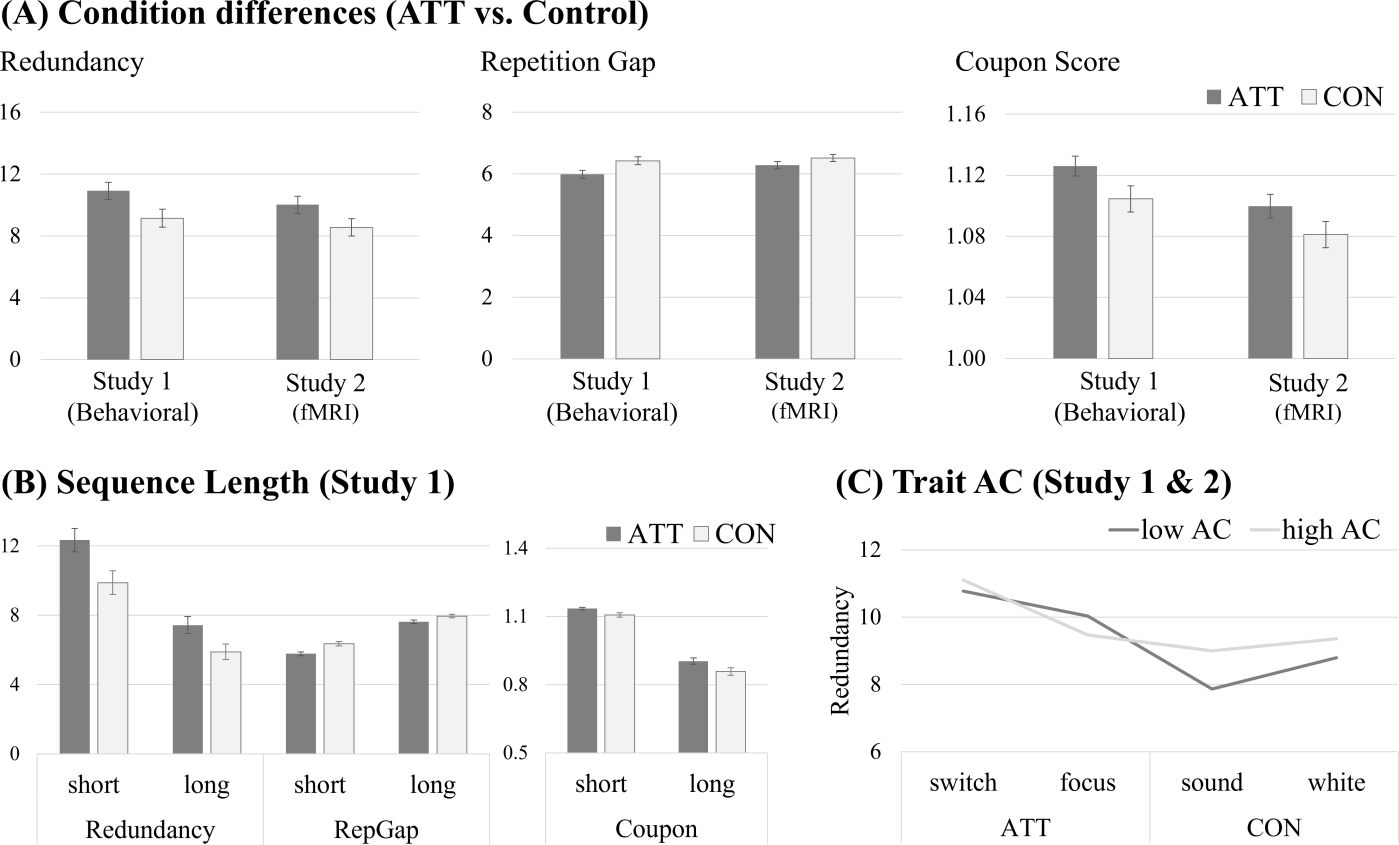
Randomness across ATT conditions. **(A)** Differences in randomness markers between ATT task conditions für Study 1 and Study 2. **(B)** Comparison of condition (ATT vs. control (CON)) differences between two task versions with longer (i.e. 45 button presses) and shorter (i.e. 21 button presses) trials. **(C)** Association between condition-dependent differences in Redundancy in subjects high and low in self-reported attentional control (AC) assessed with the Attentional Control Scale (ACS) (52). The sample was divided in high vs. low AC groups based on a median split (median score = 52) for visualization only.

A main effect of condition was detected in both studies (Study 1 (behavioral): *F*_(3, 105)_ = 14.49, *p* <.001, *η²* = .29, Study 2 (MRI): *F*_(3, 123)_ = 8.53, *p* <.001, *η²* = .17; see **Figure 2A**). *Post-hoc* tests revealed that in both studies, Redundancy was significantly higher in ATT_switch_ compared to both control conditions (*p* <.004) and in ATT_focus_ compared to CON_sound_ (*p* <.047) but not compared to CON_white_ (*p* >.145). Differences between ATT conditions were observed only in the Study 1 (behavioral) (*p* <.001) but not in Study 2 (MRI) (*p* = .132). Control conditions did not differ significantly (*p* >.058).

#### 3.1.2 Repetition gap

A main effect of condition emerged for Repetition Gap in both studies (Study 1 (behavioral): *F*_(3, 105)_ = 21.24, *p* <.001, *η²* = .38; Study 2 (MRI): *F*_(3, 123)_ = 3.94, *p* = .020, *η²* = .09; see **Figure 2A**). *Post-hoc* tests revealed that Repetition Gap was lower in ATT_switch_ compared to both control conditions in both studies (*p* <.031). Repetition Gap in ATT_focus_ was significantly lower compared to CON_sound_ in both studies (*p* <.050), but the comparison with CON_white_ reached significance only in Study 1 (behavioral) (*p* = .002), whereas no differences emerged in Study 2 (MRI) (*p* = .304). Repetition Gap was lower in ATT_switch_ compared to ATT_focus_ in Study 1 (behavioral) (*p* <.001), but not in Study 2 (MRI) (*p* = .286). No differences between control conditions were observed (*p* >.197).

#### 3.1.3 Coupon score

Coupon Scores differed between conditions in both studies (Study 1 (behavioral): *F*_(3, 105)_= 9.11, *p* <.001, *η²* = .21; Study 2 (MRI): *F*_(3, 123)_ = 3.38, *p* = .021, *η²* = .08; see **Figure 2A**). *Post-hoc* tests revealed that the Coupon Score was higher in ATT_switch_ compared to both control conditions in both studies (*p* <.012). Coupon Score in ATT_focus_ was significantly higher than in CON_sound_ in Study 1 (behavioral) (*p* <.050), whereas in Study 2 (MRI) only a trend emerged (*p* = .051). No differences were observed when comparing coupon scores between ATT_focus_ and CON_white,_ (*p* >.072). Coupon Scores were higher in ATT_switch_ compared to ATT_focus_ in Study 1 (behavioral) (*p* = .015), but this effect did not replicate in Study 2 (MRI) (*p* = .589). No differences between control conditions were observed (*p* >.511).

### 3.2 Analyses of influencing factors

#### 3.2.1 Association with standard RNG scores (sequence of 100 responses):

RNG scores in the standard RNG task were related to RNG markers during the control conditions (Redundancy: *r* = .50, *p* <.001; coupon: *r* = .29, *p* = .039) while a trend for Repetition Gap was detected (*r* = .26, *p* = .060).

#### 3.2.2 Effect of sequence length

Directly comparing randomness parameters between short and long trials in the ATT paradigm, resulted in a significant main effect of condition (Redundancy: *F*_(1, 35)_ = 29.88, *p* <.001, *η²* = .46; Repetition Gap: *F*_(1, 35)_ = 12.96, *p* <.001, *η²* = .27; Coupon Score: *F*_(1, 35)_ = 18.34, *p* <.001, *η²* = .34), with lower randomness in ATT compared to control trials (see **Figure 2B**). Further, longer trials resulted in higher randomness (Redundancy: *F*_(1, 35)_ = 142.44, *p* <.001, *η²* = .80; Repetition Gap: *F*_(1, 35)_ = 569.94, *p* <.001, *η²* = .94; Coupon Score: *F*_(1, 35)_ = 622.26, *p* <.001, *η²* = .95). A significant interaction effect between condition and trial length emerged for Repetition Gap (*F*_(1, 35)_ = 9.79, *p* = .012, *η²* = .22), with larger Repetition Gap differences in the short compared to longer trials. No interaction effects were observed for Redundancy (*F*_(1, 35)_ = 4.04, *p* = .104, *η²* = .10) or Coupon Scores (*F*_(1, 35)_ = 1.82, *p* = .186, *η²* = .05) (see **Figure 2B**).

#### 3.2.3 Association between randomness and trait AC

Descriptive information of the ACS are presented in **Table 1**. While no main or interaction effects between trait AC and neither Repetition Gap nor Coupon Scores in ATT vs. CON conditions were found (all *p* >.05), a significant interaction effect between conditions and trait AC emerged for Redundancy (*F*_(3, 72)_ = 6.43, *p* = .039, *η²* = .08). Specifically, higher trait AC was related to smaller differences in Redundancy between ATT and control trials (see **Figure 2C**).

### 3.3 Neural correlates of ATT

**Figure 3:**
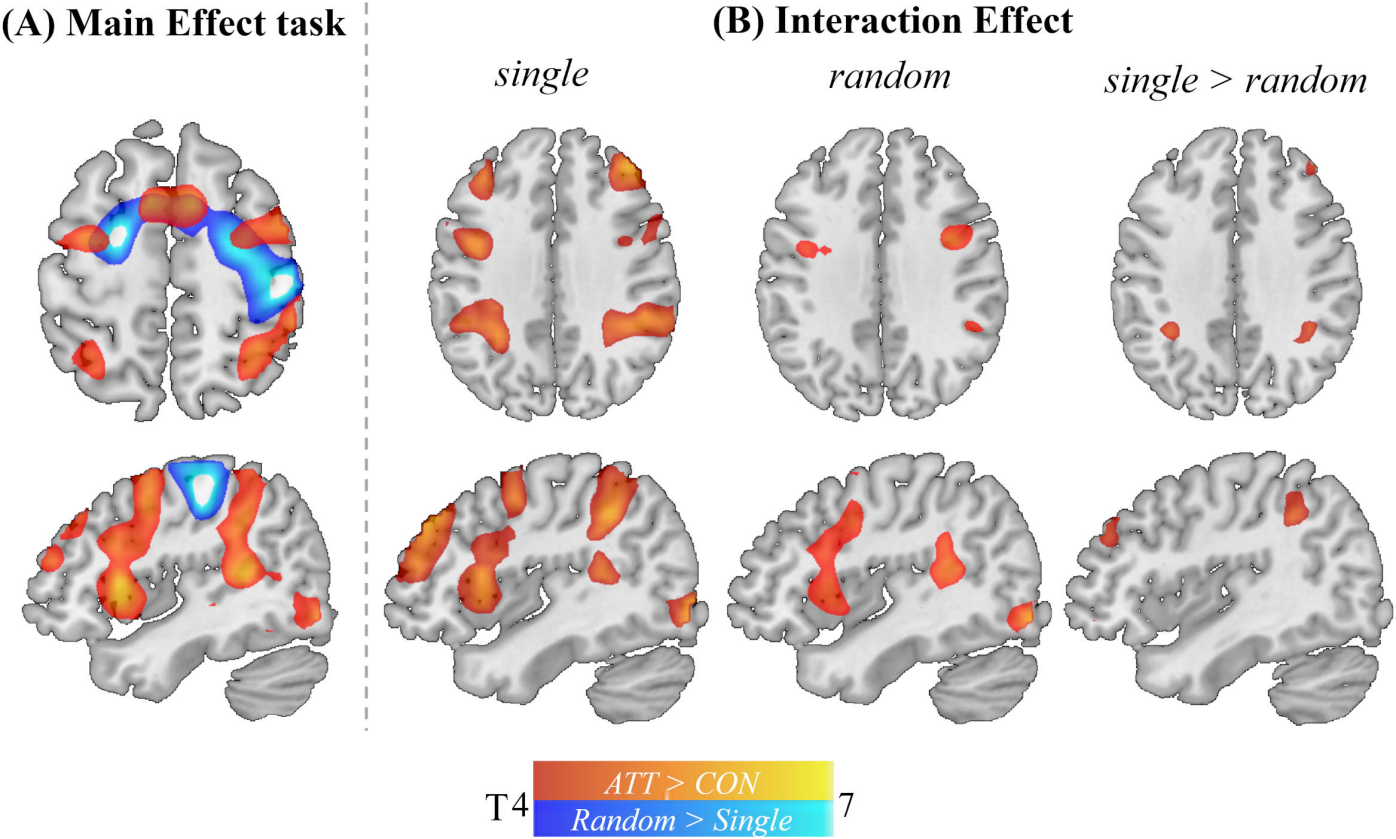
Interaction of ATT and RNG tasks. Results of one-sample t-tests contrasting ATT (combined rapid switching and focusing) versus Control (CON: combined CONsound and CONwhite) conditions (in red) and contrasting activation in random (dual-task random number generation (RNG)) versus single (dual-task single button press) conditions. For visualization only, maps were thresholded at p_uncorrected_ <.005.

In line with our recent study [same sample used in Study 2 (MRI) but without a parallel task; Schwarz et al. (21)], ATT vs. Control conditions resulted in activation of the bilateral FPN (*p_FWE_* <.05; see **Table 4**, **Figure 3A**). Independent of condition, contrasting RNG with single button presses resulted in a significantly higher whole-brain activation of the bilateral postcentral gyrus, the left lateral PFC and the left fusiform gyrus. Activation pattern highly overlapped with the dorsal attention and motor network (55) (see **Figure 3A**; **Supplementary Figure S2**; **Supplementary Table 3**). On the whole brain level, the interaction between run (RNG vs. single) and condition (ATT vs. CON) revealed no significant effect. ROI analyses resulted in a significant interaction effect in the lateral PFC (*p_FWE_* <.05, ROI corrected, see **Figure 3**; **Table 4**).

**Table 4:** Task effect (ATT > CON) in ROI analyses.

Brain region (AAL3)	MNI coordinates			*T*	*k*	*p_FWE_*
	*x*	*y*	*z*			
Dual RNG task						
R Inferior Frontal Gyrus, Triangular Part	36	17	29	6.39	100	<.001
L Precentral Gyrus	-39	-1	32	4.95	44	.005
Single button press						
R Middle Frontal Gyrus	39	44	17	7.06	254	<.001
L Middle Frontal Gyrus	-36	41	17	6.21	48	<.001
L Precentral Gyrus	-39	2	35	5.93	103	<.001
Interaction Single (ATT > CON) vs. RNG (CON > ATT)						
R Middle Frontal Gyrus	42	41	29	4.25	33	.045

Regions were classified according to the Automated Anatomical Labeling Atlas (55). Cluster extent *k* is given at *p* <.05, familywise error (FWE) corrected for multiple comparisons within the predefined left prefrontal cortex mask extracted from Glasser et al. (52) for *k*>10 voxels. *x*-, *y*-, and *z*-coordinates (MNI) and statistical information refer to the peak voxel(s) in the corresponding cluster (voxel-level statistics). R, right; L, left.

Similarly, trait AC was not significantly associated with the interaction effect between run (RNG vs. single) and condition (ATT vs. CON) on the whole brain level, but ROI analyses revealed a significant trend in the lateral PFC (*p_FWE_* = .110, ROI corrected, see **Supplementary Table 4**). Exploratory *post-hoc* tests revealed that trait AC was related to activation differences between ATT and CON conditions in the lateral PFC only in the run with a single button press task (*p_FWE_* = .035, ROI corrected), but not in the run with the parallel RNG task (*p_FWE_* = .99, ROI corrected).

## 4 Discussion

The present study investigated whether RNG could be combined with the ATT paradigm (21) to address the critical limitation that the paradigm has no behavioral measure. Specifically, we examined whether RNG performance is reduced during parallel engagement in the ATT and whether we could replicate neural effects of ATT when we combine it with a second task. For this purpose, we focused on two ATT components—selective focusing and rapid switching—which have previously been adapted in a neuroimaging design to be investigated during fMRI (16, 21). In line with our hypotheses, randomness during ATT was significantly lower compared to control conditions across three different parameters previously shown to quantify randomness in short sequences, and this effect was replicated across two independent samples. Factors such as sequence length and individual differences in AC modulated the magnitude of these effects. At the neural level, ATT was associated with activation of the FPN, while RNG was linked to activation within the dorsal attention network. However, significant interaction effects in the lateral PFC point to dual-task interference. These findings highlight the potential of RNG to capture meaningful individual differences during the ATT paradigm, although concurrent RNG performance partially masks neural task effects.

### 4.1 Reduced randomness during ATT performance

In two independent samples, we found reductions in randomness in the ATT compared to the control condition, confirming our hypothesis that attentional engagement in ATT reduces behavioral randomness across three established randomness parameters. These findings align with prior literature suggesting that deliberate engagement in controlled attentional processes – in our paradigm that is performing the ATT – constrains randomization abilities (e.g., (56). Producing a truly random sequence requires individuals to continuously monitor and regulate their responses to avoid falling back into habitual or patterned responding (57). For example, compared to truly random sequences, participants favor some options over others (i.e., Redundancy), repeat the same choice too quickly (i.e., Repetition Gap), or fail to sample all available alternatives before repeating previous choices (i.e., Coupon Score) (54). These deviations become even more pronounced when random generation is performed concurrently with another task, reflecting the high attentional demands of random responding (56). Stronger deviations from randomness in the ATT compared to the control conditions suggest that attentional resources are increasingly occupied by executing ATT. Mechanistically, ATT likely reduces randomness because continuous top-down AC consumes executive resources needed to inhibit stereotyped responses during RNG. Within the S-REF framework (5, 6), top-down executive processes, including strategic allocation of attention, govern AC. Our findings indicate that reductions in RNG during ATT reflect engagement of these executive resources, suggesting that RNG performance serves as a dynamic behavioral index of task-related AC. RNG is particularly well suited because it is simple enough to avoid ceiling effects in the secondary task which would occur in more challenging cognitive control paradigms such as the Stroop task. Further, RNG performance is relatively stable across repetitions (56, 58) making it suitable for repeated assessments throughout the entire task. Lastly, RNG is established in dual task settings targeting executive control [e.g., (43, 45, 46)].

Reductions in RNG performance during ATT were observed in two independent samples, supporting the robustness of the results. Effect sizes were generally larger in the behavioral sample (*η_p_²* = .21–.29) compared to the MRI sample (*η_p_²* = .08–.17), suggesting that contextual factors might have influenced performance. Most importantly, participants in the MRI sample had completed an additional ATT run, which may have reduced task engagement [see Schwarz et al. (21)]. In addition, the MRI environment is known to influence cognitive performance, likely due to a combination of acoustic noise, physical constraints, and increased distraction compared to standard laboratory settings (59). These factors might also explain why differences in RNG between ATT_switch_ and ATT_focus_ conditions were only observed in the behavioral Study one but not in the fMRI study. The three RNG markers were highly correlated (*r* >.60; see **Supplementary Table 1**) and differences between conditions revealed similar effect sizes. However, calculation of the Coupon Score required adaptation because many participants did not produce complete sequences within one trial probably due to the short trial lengths, leading to ceiling effects. While Angelike & Musch (53) recommended Coupon scores for short sequences, the dual-task demands in our study may have amplified the problem of incomplete sequences. Moreover, Coupon Scores showed smaller effects in both samples, suggesting that Repetition Gap or Redundancy may be more suitable measures in the ATT paradigm.

### 4.2 Analyses of influencing factors on detecting ATT-related differences in RNG performance

Besides replicating the results in an independent sample, we tested the robustness of results in additional control analyses to further explore the usefulness and validity of the dual-task RNG markers. First, to validate that RNG markers computed during ATT and control trials reflect the same construct as in standard RNG tasks which typically consist of a single sequence with a higher number of responses (usually > 100), we show that RNG markers from the standard RNG task were significantly correlated with those computed in the control conditions of the ATT paradigm. This indicates that the randomness markers obtained in the shorter, trial-wise ATT sequences capture the same underlying cognitive processes as those measured in the traditional, longer RNG task.

Second, the length of trials is a critical factor. In fMRI designs, a sufficient number of trials are needed to reliably measure task effects while not exceeding the length of the paradigm. In line with the recommended length of blocks in fMRI tasks (60), the ATT paradigm (21) consists of ~25 second blocks of ATT and control conditions. Consequently, only short sequences of random numbers can be collected in each trial (~ 20 responses), which means that RNG markers must be valid and reliable for such brief sequences. To accommodate these task constraints, we focused on three markers (i.e., Redundancy, Repetition Gap and Coupon score) recommended for assessing randomness in short sequences (54). In addition, we tested the effect of sequence length in the behavioral sample by directly comparing ATT-CON differences of RNG markers in two task versions with varying trial length (i.e., 21 vs. 43 sequences). In line with previous studies (54), randomness was higher in longer compared to shorter sequences. This means that in short sequences it is more difficult to show random behavior potentially attenuating between-condition effects. However, only for Repetition Gap we observed slightly larger condition effects for longer trials but they were also detectable in shorter trials. These results show that the short sequences of the fMRI implementation of the ATT task are sufficient to capture ATT-CON differences in RNG performance.

Third, we found that individual differences in AC influenced ATT-CON differences in one of three RNG markers. Specifically, higher trait AC was associated with smaller ATT–CON differences in Redundancy. This is consistent with previous research linking self-reported AC to both behavioral performance (52) and neural indices of AC (14, 21), suggesting that individuals with higher AC may have more resources to maintain RNG performance under the attentional load of the ATT task. Unexpectedly, however, the differences between low and high AC were only observed in the control condition, with low AC participants showing slightly better performance. No clear theoretical account fully explains this pattern. Therefore, it may also be attributable to methodological factors such as sampling variability, measurement noise, or chance effects. These findings should be interpreted cautiously and warrant replication.

Overall, these control analyses demonstrate the robustness and validity of the dual-task RNG markers. RNG performance during the ATT paradigm is comparable with standard RNG tasks, is sensitive to detect differences between ATT and control conditions even with shorter trial sequences, and are related to intraindividual differences in AC.

### 4.3 Neural correlates of the adapted ATT paradigm

At the neural level, our findings partly replicate and extend prior work (16, 21). The ATT conditions elicited higher activation in the bilateral FPN than the control conditions, consistent with our earlier study without the concurrent RNG task (21) and previous investigations of the neural correlates of the ATT (15, 16). Furthermore, RNG compared to single button presses showed stronger activation in the dorsal parts of the frontal cortex, particularly in the frontal eye fields which is in line with existing fMRI studies [e.g., (36–41)]. This pattern likely reflects increased demands on sustained attentional orienting and top-down selection, as participants must continuously inhibit prepotent response tendencies while actively monitoring and updating response sequences. The engagement of the frontal eye fields, a core node of the dorsal attention network (61), suggests that RNG relies on goal-directed allocation of attentional resources to maintain response variability (37). Thus, our data show, that RNG recruits AC via the dorsal attention network.

Directly comparing the networks engaged by RNG and ATT reveals distinct activation in separable functional networks (see **Figure 3A**). However, ROI analyses showed interaction effects in the lateral inferior PFC, pointing to interference between ATT and RNG performance at the neural level. These results resonate with earlier work showing that dual-task paradigms combining RNG with other cognitive challenging tasks produce interference effects [e.g., (43)]. Importantly, a recent meta-analysis revealed that dual-task performance leads to activation in highly similar regions of the FPN (i.e. IPS and inferior frontal cortex) targeted by our ATT paradigm (62), suggesting that activation in this network reflects domain-general cognitive control mechanisms necessary for coordinating multiple task demands. Thus, shared engagement of the FPN by ATT and RNG or the execution of multiple tasks simultaneously may have led to shared variance, thereby reducing statistical power to detect condition differences on the neural level. This interpretation is further supported by the associations between neural ATT-control differences and trait AC during an easy task (i.e. single button press) but not during the more demanding RNG task. This association in the single button task is consistent with our recent study (21) where we showed that left lateral PFC activation during ATT (without a dual-task) related to self-reported AC. Note that this previous finding was examined in the same sample reported here but in a separate run of the ATT paradigm without a parallel task (21). Although these exploratory effects are weak and require replication in an independent dataset, we observed an attenuation of condition effects at the neural level and associations with trait AC disappeared with concurrent RNG suggesting that investigating the neural correlates of ATT might be impeded by combining ATT with RNG.

### 4.4 Limitations

Taken together, our results demonstrate that RNG is a feasible and informative parallel task for quantifying the degree of engagement in the ATT paradigm. Nonetheless, several limitations warrant consideration. First, our paradigm differed from the original ATT by using discrete, randomized blocks and omitting the divided attention condition, which was originally brief and previously shown to have minimal impact on brain responses (16). Nonetheless, given evidence that this component may play a relevant role (17, 20), future studies should examine ATT subcomponents more systematically, ideally within-subject. Second, the absence of standardized definitions of randomness complicates interpretation, as many different measures exist and may capture distinct aspects of behavior [e.g., (54, 63, 64)]. Third, recent studies show that specific instructions influence RNG behavior, with explicit versus implicit framing affecting performance (15, 65). In our paradigm, participants received concurrent ATT and RNG instructions, with visual cues prompting key presses. Slight differences in how participants interpreted and implemented these instructions—whether prioritizing ATT performance or balancing both tasks—may have affected both RNG performance and the neural responses observed for ATT subcomponents [e.g., (66)]. Fourth, RNG behavior also reflects stable, person-specific strategies (15), making it a sensitive index of attentional engagement, but one that is influenced by individual differences. In particular, factors such as executive dysfunction, state anxiety, or worry are known to reduce RNG performance (49, 50), which, while interesting in itself, may interfere with detecting ATT-specific effects. Building on these limitations, in future work we plan to investigate independent, more indirect markers of engagement, such as pupil dilation differences between ATT and control trials, to quantify AC during ATT more robustly.

## 5 Conclusion

The present study shows that engagement in ATT reduces randomness in behavior, with effects robustly shown in two independent samples and different task versions. This supports the theoretical notion that ATT recruits and occupies AC resources, thereby constraining performance on concurrent tasks that rely on executive functions. Further, associations between task-related differences in one of the three RNG markers related to trait AC, highlighting the relevance of these measures for capturing individual differences in AC. While neural correlates replicate previous effects of ATT and RNG, results point towards an interference by the RNG task in lateral PFC regions. This interference represents a potential limitation for the use of RNG as a behavioral marker of ATT engagement, particularly when focusing on neural correlates which calls for testing alternative, behavior-independent measures of ATT engagement. Overall, reductions in randomness provide a quantifiable, performance-based measure of ATT engagement that complements self-report measures and may help monitor training progress or treatment response in clinical settings.

## Data Availability

TThe data that support the findings of this study are available in the Open Science Framework (OSF) at https://osf.io/7q25a/overview.
