# Supplementary material for: Randomness as a behavioral marker of attentional control during the attention training technique and its neural correlates
Source: Front Psychiatry. 2026 Apr 24;17:1765858. doi: 10.3389/fpsyt.2026.1765858 (PMC13154155; doi:10.3389/fpsyt.2026.1765858)
Supplement: Supplementary file 1 [file SupplementaryFile1.docx]

***Supplementary Material***

**Randomness as a Behavioral Marker of Attentional Control during the Attention Training Technique and its neural correlates**

1. **ROI mask**

**Figure S1: Region-of-Interest mask**

**
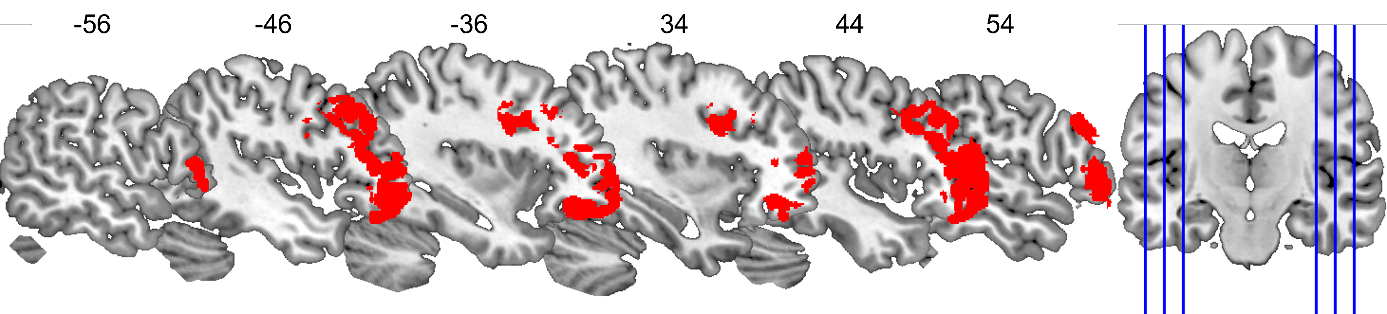
**

1. **Correlation between RNG markers**

**Table S1.** Correlation between RNG markers (N=75)

|  |  | | **Coupon Score** | | **Redundancy** | | **Repetition Gap** | |
| --- | --- | --- | --- | --- | --- | --- | --- | --- |
|  |  | | **ATT** | **CON** | **ATT** | **CON** | **ATT** | **CON** |
| Coupon Score | ATT | r | 1.000 |  |  |  |  |  |
|  | CON | r | .741** | 1.000 |  |  |  |  |
| Redundancy | ATT | r | .820** | .680** | 1.000 |  |  |  |
|  | CON | r | .638** | .774** | .796** | 1.000 |  |  |
| Repetition Gap | ATT | r | -.707** | -.649** | -.786** | -.694** | 1.000 |  |
|  | CON | r | -.597** | -.761** | -.664** | -.869** | .747** | 1.000 |
| *Note*: * p<.05; **p<.001; pearson partial correlation (corrected for sample: 1: behavioral; 2: MRI sample). RNG: Random number generation; ATT: Attention Training Technique; CON: control. | | | | | | | | |

1. **fMRI results**

**Table S2:** Task effect (ATT > CON) in whole brain analyses

| Brain region (AAL3) | MNI coordinates | | | *T* | *k* | *p_FWE_* |
| --- | --- | --- | --- | --- | --- | --- |
|  | *x* | *y* | *z* |  |  |  |
| *Random* |  |  |  |  |  |  |
| R Superior Temporal Gyrus | 54 | -19 | 2 | 10.6 | 1024 | <.001 |
| L Superior Temporal Gyrus | -45 | -43 | 17 | 9.7 | 878 | <.001 |
| R Inferior Occipital Gyrus | 33 | -91 | -7 | 8.62 | 74 | <.001 |
| L Inferior Occipital Gyrus | -21 | -100 | -7 | 7.65 | 94 | <.001 |
| L Inferior Frontal Operculum | -36 | 17 | 14 | 7.29 | 90 | <.001 |
| R Insula | 36 | 20 | 14 | 6.64 | 185 | .001 |
| R Precentral Gyrus | -36 | -4 | 44 | 5.72 | 12 | .013 |
| *Single* |  |  |  |  |  |  |
| R Superior Temporal Gyrus | 60 | -34 | 11 | 9.92 | 1478 | <.001 |
| L Superior Temporal Gyrus | -51 | -19 | 8 | 9.74 | 867 | <.001 |
| L Supplementary Motor Area | -9 | 14 | 47 | 7.16 | 154 | <.001 |
| L Inferior Frontal Operculum | -39 | 11 | 17 | 7.11 | 183 | <.001 |
| R Middle Frontal Gyrus | 39 | 44 | 17 | 7.06 | 243 | <.001 |
| L Precentral Gyrus | -42 | -4 | 38 | 6.52 | 114 | .001 |
| L Middle Frontal Gyrus | -36 | 38 | 17 | 6.51 | 66 | .001 |
| L Lobule VIII of Cerebellar Hemisphere | -27 | -70 | -52 | 6.41 | 40 | .002 |
| R Precentral Gyrus | 36 | 0 | 41 | 6.05 | 50 | .005 |
| L Inferior Occipital Gyrus | -27 | -94 | -7 | 5.95 | 34 | .007 |
| R Inferior Occipital Gyrus | 42 | -82 | -7 | 5.85 | 35 | .009 |
| R Precentral Gyrus | 30 | -7 | 53 | 5.57 | 12 | .020 |
| *Interaction Single (ATT > CON) vs. Random (CON > ATT)* | | | | | |  |
| No results |  |  |  |  |  |  |
| *Note*: Regions were classified according to the Automated Anatomical Labeling Atlas (1). Cluster extent *k* is given at *p* < .05, familywise error (FWE) corrected for multiple comparisons within the whole brain for *k*>10 voxels. *x*-, *y*-, and *z*-coordinates (MNI) and statistical information refer to the peak voxel(s) in the corresponding cluster (voxel-level statistics). R, right; L, left. | | | | | | |

**Table S3:** Main effect of run (random > single) in whole brain analyses

| Brain region (AAL3) | MNI coordinates | | | *T* | *k* | *p_FWE_* |
| --- | --- | --- | --- | --- | --- | --- |
|  | *x* | *y* | *z* |  |  |  |
| R Postcentral Gyrus | 48 | -25 | 50 | 9.42 | 509 | <.001 |
| L Superior Frontal Gyrus | -21 | -4 | 56 | 9.20 | 249 | <.001 |
| L Postcentral Gyrus | -42 | -34 | 44 | 7.48 | 61 | <.001 |
| L Fusiform Gyrus | -27 | -73 | -7 | 6.64 | 45 | .001 |

*Note*: Regions were classified according to the Automated Anatomical Labeling Atlas (1). Cluster extent *k* is given at *p* < .05, familywise error (FWE) corrected for multiple comparisons within the whole brain for *k*>10 voxels. *x*-, *y*-, and *z*-coordinates (MNI) and statistical information refer to the peak voxel(s) in the corresponding cluster (voxel-level statistics). R, right; L, left.

**Figure S2:** Overlap between activation in the random > single contrast (blue) and the dorsal attention network (green)


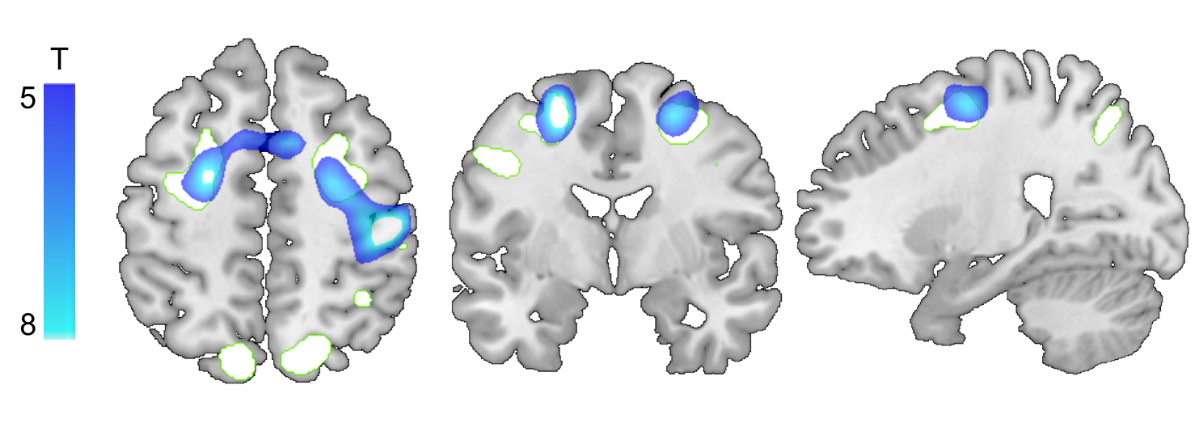


Blue: Results of one-sample t-tests contrasting random (dual-task random number generation) versus single (dual-task single button press) conditions. Green: dorsal attention network according to Glasser and colleagues (2).

**Table S4:** Association ATT>CON with trait AC in ROI analyses

| Brain region (AAL3) | MNI coordinates | | | *T* | *k* | *p_FWE_* |
| --- | --- | --- | --- | --- | --- | --- |
|  | *x* | *y* | *z* |  |  |  |
| *Random* |  |  |  |  |  |  |
| No results |  |  |  |  |  |  |
| *Single* |  |  |  |  |  |  |
| L Middle Frontal Gyrus* | -33 | 56 | 14 | 4.38 | 11 | .035 |
| *Interaction Single (ATT > CON) vs. Random (CON > ATT)* | | | | | | |
| L Middle Frontal Gyrus* | -33 | 53 | 11 | 3.93 | 12 | .110 |

*Note*: Regions were classified according to the Automated Anatomical Labeling Atlas (1). Cluster extent *k* is given at *p* < .05, familywise error (FWE) corrected for multiple comparisons within the predefined left prefrontal cortex mask extracted from Glasser et al., (2) for *k*>10 voxels. *x*-, *y*-, and *z*-coordinates (MNI) and statistical information refer to the peak voxel(s) in the corresponding cluster (voxel-level statistics). R, right; L, left.

**References**

1. Rolls ET, Huang CC, Lin CP, Feng J, Joliot M. Automated anatomical labelling atlas 3. Neuroimage. 2020 Feb 1;206:116189.

2. Glasser MF, Coalson TS, Robinson EC, Hacker CD, Harwell J, Yacoub E, et al. A multi-modal parcellation of human cerebral cortex. Nature 2016 536:7615 [Internet]. 2016 Jul 20 [cited 2024 Apr 25];536(7615):171–8. Available from: https://www.nature.com/articles/nature18933
